# Systematic HPLC/ESI-High Resolution-qTOF-MS Methodology for Metabolomic Studies in Nonfluorescent Chlorophyll Catabolites Pathway

**DOI:** 10.1155/2015/490627

**Published:** 2015-02-09

**Authors:** José Julián Ríos, María Roca, Antonio Pérez-Gálvez

**Affiliations:** Food Phytochemistry Department, Instituto de la Grasa, Consejo Superior de Investigaciones Científicas (CSIC), Pablo de Olavide University Campus, Carretera de Utrera Km 1, 41013 Sevilla, Spain

## Abstract

Characterization of nonfluorescent chlorophyll catabolites (NCCs) and dioxobilane-type nonfluorescent chlorophyll catabolite (DNCC) in peel extracts of ripened lemon fruits (*Citrus limon* L.) was performed by HPLC/ESI-high resolution-qTOF-MS method. Compounds were identified in samples on the basis of measured accurate mass, isotopic pattern, and characteristic fragmentation profile with an implemented software postprocessing routine. Three NCC structures already identified in other vegetal tissues were present in the lemon fruit peels (*Cl*-NCC1; *Cl*-NCC2; *Cl*-NCC4) while a new structure not defined so far was characterized (*Cl*-NCC3). This catabolite exhibits an exceptional arrangement of the peripheral substituents, allowing concluding that the preferences for the NCC modifications could be a species-related matter.

## 1. Introduction

In senescent leaves and in ripened fruits, chlorophylls are catabolised into terminal chlorophyll catabolites named nonfluorescent chlorophyll catabolites (NCC) and dioxobilin-type NCC (DNCC) that are accumulated into the vacuoles [1]. Since they were identified for the first time [2, 3], few different structures (13 NCC and 7 DNCC, Table 1) have been described.

**Figure 1:**
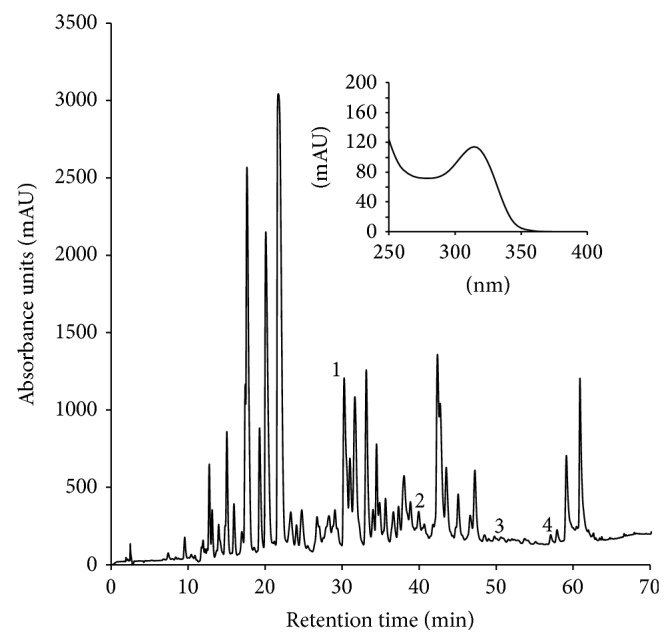
HPLC chromatogram of lemon fruit peels extract acquired at 320 nm, showing the target compounds described in Table 2. 1:* Cl*-NCC1; 2:* Cl*-NCC2; 3:* Cl*-NCC3; 4:* Cl*-NCC4. Insert is the UV-vis spectrum of peak 2.

All NCC/DNCC derives, through an enzymatic cascade, from chlorophyll molecule, having a common structure (Table 1), with the porphyrin macrocycle cleaved at the C(4):C(5) mesoposition. All three remaining mesopositions are fully reduced, resulting in the complete disruption of the double-bonding system of chlorophylls. NCCs are nonfluorescent and colorless with a maximum of absorption at 315 nm (see insert in Figure 1) which is due to the *α*-formyl pyrrole moiety of ring B [4]. Due to the high selectivity in the functionalization reaction(s) of the precursor that originate NCCs, only three positions are allowed to be modified with defined groups, as follows (Table 1): at C3, the initial vinyl group can be dihydroxylated or not; at C8^2^ a hydrogen, a hydroxyl, a glucopyranosyl, a malonyl, or a O-malonyl-glucopyranoside group has been described; and finally O13^4^ can be methylated or not [5]. The other chlorophyll catabolite, DNCC, the dioxobilin-type NCC, lack the formyl group at C6, a function presents in all NCCs (Table 1) [6] and consequently the corresponding band at 315 nm. Up to now, the same functional groups described for NCCs are found in the DNCCs.

A convention for the nomenclature of NCC/DNCC was established by Ginsburg and Matile [7] taking the initials of the latin species name in which the compound is described, followed by the abbreviation “NCC or DNCC” with a number which indicates elution order in the HPLC analysis. Consequently, several NCC/DNCC structures present different names depending on the species where they have been identified, although they are the same compound. Probably, the more NCC/DNCC structures are identifed, the more need of a practical nomenclature system is necessary. NMR has been the analytical approach applied to identify the molecular structure of NCC and DNCC [8, 9]. Indeed, taking into account the quantities existent in a tissue, only the chlorophyll catabolites present in high amounts have been identified by NMR, complemented in most of the cases with the application of mass spectrometry to obtain spectroscopic information of the molecules but not with the aim of screening. With such analytical approach, much information is lost, avoiding the acquisition of results regarding chlorophyll metabolism. Exclusive application of mass spectrometry has not been the single selected strategy until recently [10–12] for NCC/DNCC characterization, considering the elucidated NCC and DNCC structures in several vegetal tissues what has allowed the achievement of valuable knowledge about their chemical behaviour. MS^2^ fragmentation pattern is available in most of the structures already identified, with the advantage that only few arrangements (substituents at the peripheral positions) are possible, what facilitates structural identification of already known NCC/DNCC in new explored vegetal tissues and even the prediction of unanticipated structures. Consequently, new strategies are necessary to study the complexity of reactions that can experiment the chlorophyll catabolites in leaves and fruits. Such methodologies include DESI-MS [10], ESI-MS [13], and ESI-IT-MS^2^ analysis with powerful postprocessing software [11, 12]. For example, Target Analysis has been successfully applied for screening of some other phytochemicals [14, 15].

*Citrus* fruits and their products are highly demanded through the world. The fruits present a wide arrange of internal and external colours as a consequence of several factors with different influence in the existing colour range, including natural evolution and agronomic selection [16]. Thus, the diversity of fruit peel colouration ranges from green of limes to pink in red grapefruits, orange in mandarins and sweet oranges, and yellow in lemons. However, all of the fruits share that their external coloration is green at the immature stage due to the presence of chlorophylls and that these pigments start to decrease during ripening [17] to be almost absent at the mature stage, as a consequence of the chlorophyll catabolism. Therefore, fruits of* Citrus* species could be a suitable tissue for the analysis of terminal chlorophyll catabolites (NCC and DNCC). And this was the aim of this work, to analyse the profile of chlorophyll catabolites in the peel of ripened lemon fruits considering the compounds already identified in different vegetal species (leaf and fruit tissues) and some other structures that, following the structural constraints mentioned above are possible. This objective was achieved with the application of HPLC-high resolution ESI-qTOF-MS in combination with MS^2^-based reactions and with the use of an automatic formula determination routine to increase the analytical efficiency and the high specificity achieved from MS data.

## 2. Materials and Methods

### 2.1. Plant Material

Lemon fruits (*Citrus limon* L.) were purchased in a local market. Determination of the NCC/DNCC profile was performed in naturally yellow fruits. Maize (*Zea mays* L.) seeds were germinated to harvest primary leaves, while leaves of spinach (*Spinacea oleracea* L.) were bought at local market. They were allowed to senescence by dark-incubation in distilled water in Petri dishes at 25°C during 5 to 7 days [18].

### 2.2. Reagents

Potassium phosphate was provided by Sigma-Aldrich Chemical Co. (Madrid, Spain). HPLC LC/MS grade solvents were supplied by Panreac (Barcelona, Spain). The deionized water used was obtained from a Milli-Q 50 system (Millipore Corp., Milford, MA, USA). Na (NaCOOH) (10 mM NaOH in 300 *μ*L of formic acid) was used for calibration.

### 2.3. NCC/DNCC Extraction

Fresh material (peels of ripened lemon fruits, senescent leaves of spinach and maize) was homogenized in liquid nitrogen and extracted into 10 volumes of acetone during 30 min in a vortex at maximum speed. The extract was centrifuged at 14000 ×g for 5 min and the pellet was extracted again. Supernatants from both extractions were joined and concentrated in a rotary evaporator. The aqueous residue was partially diluted with MeOH (20%) and applied to a SPE column (C_18_, Bakerbond SPE, 500 mg/6 mL, J. T. Baker, Deventer, Holland) activated with two volumes of methanol and two volumes of water. The SPE with the sample was cleaned with four volumes of water for desalting, and the NCCs fraction was eluted with 1 mL of 20 mM KPi pH 7.0/methanol (1 : 9, v/v) [8, 19, 20].

### 2.4. Liquid Chromatography/Electrospray Ionization Ion Trap/Time-of-Flight Mass Spectrometry

The liquid chromatograph system was Dionex Ultimate 3000 RS U-HPLC (Thermo Fisher Scientific, Waltham, MA, USA). Chromatographic separation was performed as described earlier [21] but with modifications as follows. The eluent components were 0.1% (v/v) formic acid in water (A) and 0.1% (v/v) formic acid in methanol (B). The proportion of B was increased from 20% to 32% in 10 min, to 40% in 15 min, to 60% in 30 min, and to 100% in 10 min and held at 100% for 18 min. Initial conditions were reached in 2 min and the equilibrium time was 10 min. The injection volume was 30 *μ*L and the flow rate was 1 mL/min. A stainless steel column (20 × 0.46 cm i.d.), packed with 3 *μ*m C_18_ Spherisorb ODS-2 (Teknokroma, Barcelona, Spain), was used. A split post-column of 0.4 mL/min was introduced directly on the mass spectrometer electrospray ion source. The HPLC/ESI-qTOF operated for mass analysis using a micrOTOF-QII High Resolution Time-of-Flight mass spectrometer (UHR-TOF) with qQ-TOF geometry (Bruker Daltonics, Bremen, Germany) equipped with an electrospray ionization (ESI) interface. This equipment was used to obtain the high resolution MS data and obtain a first tentative candidate list of NCC/DNCC. The HPLC/ESI-IT system operated for MS^2^ analyses, selecting each* m*/*z* value of the protonated ions of the first tentative candidate list of NCC/DNCC to attain exclusively its product ions and obtain a second candidate list of NCC/NDCC. In this case, the instrument was operated in positive ion mode using a scan range from* m*/*z* 50 to 1200. The HPLC/ESI-qTOF-MS^2^ analyses were performed to check authenticity of product ions identified by the HPCL/ESI-IT and obtain the final identified list of NCC/NDCC. Mass spectra were acquired in MS fullscan mode and data were used to perform multitarget-screening using TargetAnalysis 1.2 software (Bruker Daltonics, Bremen, Germany). MS^2^ spectra were acquired in Auto-MS^2^ mode (data-dependent acquisition) and were used for structural confirmation of compounds detected. Collision energy was estimated dynamically based on appropriate values for the mass and stepped across a ±10% magnitude range to ensure good quality fragmentation spectra. The instrument control was performed using Bruker Daltonics HyStar 3.2.

### 2.5. Data Analysis

An* in-house* mass database created* ex professo* comprises monoisotopic masses, elemental composition and, if known, retention time and characteristic fragment ions, for 13 NCCs and the seven DNCCs [3, 8, 9, 11, 12, 19, 21–29]. Additionally, elemental composition of new NCCs/DNCCs not described so far was included in the database. These new chlorophyll catabolites were generated holding two rules: only those substituents already described in literature are allowed to enter in the basic NCC/DNCC structure and in the same carbon atom positions; dioxobilin-type chlorophyll catabolites are generated from all NCCs structures (both known and new ones) by deformylation at C6 in ring B. For example, a new proposed NCC structure is the deesterified form of* Nr*-NCC1. Data evaluation was performed with Bruker Daltonics DataAnalysis 4.0. From the HPLC/TOF-MS acquisition data, an automated peak detection on the EICs expected for the [M + H]^+^ ions of each compound in the database was performed with Bruker Daltonics TargetAnalysis 1.2 software. The software performed the identification automatically according to mass accuracy and in combination with the isotopic pattern in the SigmaFit algorithm. This algorithm provides a numerical comparison of theoretical and measured isotopic patterns and can be utilized as an identification tool in addition to accurate mass determination. The calculation of mSigma values includes generation of the theoretical isotope pattern for the assumed protonated molecule and calculation of a match factor based on the deviations of the signal intensities. Only those hits with mass accuracy and mSigma values within the tolerance limits, which were set at 5 ppm and 50, respectively, are included in the final report list that was carried out using a Microsoft EXCEL-based script. This software has been successfully applied for screening of NCC/DNCC [11, 12] and other phytochemicals [14, 15]. The interpretation of the MS^2^ spectra was performed using the SmartFormula3D module included in the DataAnalysis software. This module includes an algorithm that estimates whether a formula for a product ion is a subset of a formula for the precursor. Based on expected chemistry, elements carbon, hydrogen, oxygen, nitrogen, bromine, and iodine were allowed. Sodium and potassium were also included for the calculation of adduct masses. The number of nitrogen atoms was limited to an upper threshold of ten. The number of rings plus double bonds was checked to be chemically meaningful (between 0 and 50). For each NCC/DNCC detected in the sample, the module shows the original MS and MS^2^ data as peak lists. From all possible formulae for the precursor ion, only one should fit with the elemental composition expected for the protonated molecule and satisfy thresholds for mass accuracy and mSigma values. Once the correct formula is selected, the module displays the formulae and neutral losses in the MS^2^ spectrum fitting to the boundary conditions for the precursor ion, and they should be consistent with the MS^2^ data peak list. The SmartFormula3D checks the consistency highlighting the monoisotopic peaks with formula suggestion and the related isotopic peaks. Based on this combined data evaluation, fragmentation pattern for each NCC/DNCC can be generated to support its identification in the sample.

Mass Frontier 4.0 is a software package for the management, evaluation, and interpretation of mass spectra, including the automated generation of possible fragments and rearrangement mechanisms, starting from a user-supplied chemical structure. With this feature of the software we can check consistency between a chemical structure and its mass spectrum and recognize the structural differences between spectra of closely related compounds. The program generates a fragmentation scheme for the drawn molecular structure using fragmentation rules of mass spectrometry known in the literature, as well as the selected ionization mode and the number of fragmentation steps. The program parameters used in this study were ESI ionization method, inductive cleavage and 5 as maximum number of reaction steps. The fragmentation reactions were selected to include hetero and homolytic cleavage, neutral losses, and hydrogen rearrangements. Other parameters were left as their default values.

## 3. Results and Discussion

**Figure 2:**
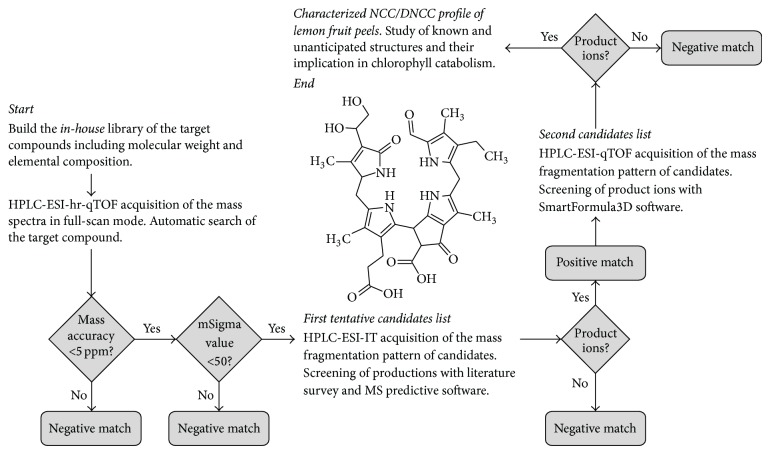
Data evaluation routine of HPLC/qTOF-MS data: to build an* in-house* library of elemental composition of the target compounds; automated peak detection on the EICs expected for the [M + H]^+^ ions of each compound in the* in-house* library. The software performs the identification automatically according to mass accuracy and in combination with the isotopic pattern in the SigmaFit algorithm. A first tentative candidates list is obtained. To acquire IT-MS^2^ fragmentation pattern of tentative candidates to confirm structural configuration in comparison with literature data and predictive software. A second candidates list is obtained. Acquisition of high resolution ESI-qTOF-MS^2^ data to confirm consistency of product ions and to obtain the final characterized NCC/DNCC profile.

A careful evaluation of metabolite content in a defined biological system is needed because of the high degree of chemical diversity. Two general analytical platforms provide complementary information on the analysis of complex mixtures: NMR and MS. Chemical structure and quantity are frequently characterized by NMR but its capacity is limited to most abundant compounds in a sample. MS techniques are many orders of magnitude more sensitive and can detect many more compounds per a unit of time. In this work, the high resolution TOF-MS acquired data were matched and correlated with an* in-house* database of specific metabolites (NCC and DNCC) containing their exact monoisotopic masses, calculated from their molecular formula. This database was built from the data published regarding NCC and DNCC identification in vegetal tissues and encloses 20 (13 NCCs and 7 DNCCs) different molecular formula shown in Table 1, including their molecular weight and elemental composition as well as their name following the nomenclature convention [7]. Two criteria were used for compound identification, accurate mass and the mSigma value obtained with the SigmaFit algorithm, an orthogonal criterion for compound identification independent of mass measurements, which indicates the agreement between the theoretical and the measured isotopic pattern of the mass signal of interest. Only those hits with mass accuracy and mSigma values within the tolerance limits, which were set at 5 ppm and 50, respectively, are included in the positive matching list.  Figure 2 shows the flow of the systematic approach used for screening and positive matching the NCC/DNCC present in lemon fruit peels.

This survey approach was complemented with the evaluation of the MS^2^-based reaction of the protonated molecules positively identified with the above described criteria. To achieve this, extracts of lemon peels were analysed by HPLC-IT-MS^2^, obtaining the MS^2^ fingerprint. The characteristic fragment ions of the matched compound were compared with those of authentic NCC/DNCC standards, if the first was one of the already chlorophyll catabolites described in literature. When the matched compound was an unanticipated species then the fragmentation profile was compared with the one obtained from Mass Frontier, a rule-based prediction software of fragment ions from a given candidate molecule. Additionally, HPLC-UV-visible data (retention time and UV-vis. spectra) were used to definitively evaluate the NCC/DNCC composition of lemon fruit peels.

Figure 1 shows the HPLC chromatogram at 320 nm of an extract of lemon fruit peels, where 4 NCCs were identified. In some cases it was possible to observe the typical UV spectrum of NCCs with a maximum at 314 nm, characteristic of an *α*-formylpyrrole moiety at ring B [4]. This observation was complemented with the survey approach described above and the results are shown in Table 2 that includes the list of positive matches, their elemental composition, experimental exact mass, retention time, mass error, and mSigma value. It is noteworthy that any DNCC has been identified in lemon fruit peels, in spite that the complete list of already identified DNCCs is included in the postprocessing software. In addition, and as a systematic methodology applied in this research, from all the already NCC known, the corresponding standards were obtained from the appropriate raw material. Therefore, it was possible to check the coelution and the coincidence of the fragmentation pattern after IT-MS^2^ analysis between the standard and the preliminary identified NCC obtained from lemon fruit peels.

**Figure 3:**
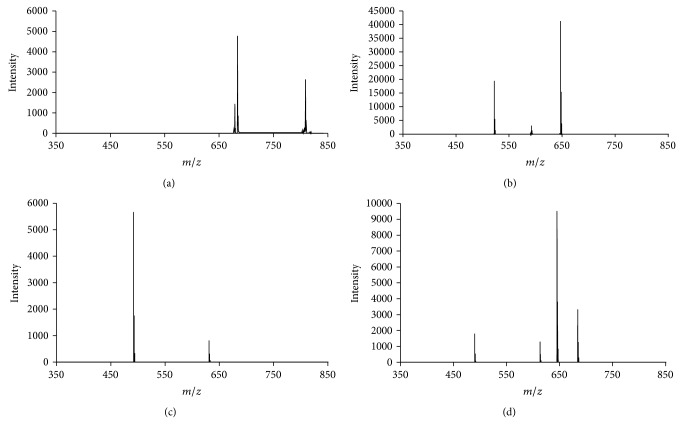
High performance liquid chromatography IT-MS^2^ ion spectra of (a)* Cl*-NCC1 (*m*/*z* 841.3), (b)* Cl*-NCC2 (*m*/*z* 679.2), (c)* Cl*-NCC3 (*m*/*z* 649.2), and (d)* Cl*-NCC4 (*m*/*z* 807.3) in peels of lemon fruits extracts. Product ions are detailed in Table 2.

The first NCC identified in lemon fruit peels is the* Cl*-NCC1 (31.6 min, peak 1, Figure 1), following the nomenclature convention established by Ginsburg and Matile [7]. It shows an accurate mass of* m*/*z* 841.3473 and a predicted molecular formula of C_41_H_52_N_4_O_15_, identical to that of the compound previously identified in senescent leaves of maize (*Zm*-NCC1; [23]),* Tilia Cordata* Mill. (*Tc*-NCC1; [24]) and quince fruits (*Co*-NCC1; [11]).* Cl*-NCC1 presents a dihydroxylated vinyl group at C3, a glucosyl moiety at C8^2^ and methyl group at O13^4^ (see Table 2). IT-MS^2^ fragmentation of* Cl*-NCC1 is replicated in the corresponding analysis of the standard* Zm*-NCC1 and detailed in Table 2. In fact, the MS^2^ analyses of both NCCs show the same fragmentation pattern: signals at* m*/*z* 809, 684, and 679, which correspond to the loss from the protonated molecule of MeOH, as it is typical of methyl ester NCCs, to the loss of ring A, and to the loss of the sugar moiety as [C_6_H_10_O_5_].  Figure 3(a) depicts the IT-MS^2^ analysis corresponding to this compound. All these product ions are characteristic losses of* Zm*-NCC1/*Tc*-NCC1/*Co*-NCC1 [11, 23, 24]. In addition, we made the high-resolution ESI-qTOF-MS^2^, analysis of* Zm*-NCC1 in maize and* Cl*-NCC1 in lemon fruit peels to confirm the consistency of the elemental composition and formula of all product ions, obtaining positive records in all cases. Therefore,* Cl*-NCC1 is the equivalent of* Zm*-NCC1/*Tc*-NCC1/*Co*-NCC1 in lemon fruit peels.

The second NCC identified in lemon fruit peels is* Cl*-NCC2 (39.5 min, peak 2, Figure 1) with an accurate mass obtained with high resolution ESI-qTOF-MS of* m*/*z* 679.2953, and a predicted molecular formula of C_35_H_42_N_4_O_10_. The same molecular mass and elemental composition have been previously described for a tentative NCC analogue in senescent leaves of barley (*Hv*-NCC1) by Kraütler et al. [25] and in senescent spinach leaves (*So*-NCC2) by Oberhuber et al. [8]. To confirm the equivalence of* Cl*-NCC2 with the NCC previously described in senescent leaves of spinach we obtained an extract from senescent freshly harvested spinach leaves, which was analysed with the experimental conditions described here. The compound from lemon fruit peels (*Cl*-NCC2) and the* So-*NCC2 coeluted at the same retention time. The literature describes for* Hv*-NCC1/*So*-NCC2 after MS^2^ fragmentation a typical signal at 522, as a consequence of the ring A loss. To characterize* Cl*-NCC2, the IT-MS^2^ analysis was performed, obtaining the characteristic signals at* m*/*z* 647 which correspond to the loss of MeOH from the protonated molecule, as it is typical of the methyl ester functionality, and* m*/*z* 522 corresponding to the loss of ring A (see Figure 3(b)). Consistency of the elemental composition and formula of all product ions was checked with the SmartFormula3D algorithm by high-resolution ESI-qTOF-MS^2^ analysis of the* Cl*-NCC2 observed in lemon fruit peels and* So*-NCC2 in spinach. Taking in consideration all these results,* Cl*-NCC2 presents at C3 a dihydroxylated vinyl group, at C8^2^ a hydroxyl group, and a methyl group at O13^4^ (see structure in Table 2).

The third chlorophyll catabolite detected in lemon fruit peels is* Cl*-NCC3 (51.6 min, peak 3, Figure 1) which exhibits the typical UV-spectrum of a NCC. The protonated molecule of this unanticipated product was observed at* m*/*z* 649.2852 in the high-resolution ESI-qTOF-MS with mass error and mSigma values within the threshold limits (see Table 2). This compound presents an elemental composition C_34_H_40_N_4_O_9_ that considering the structural skeleton of NCCs and the described functional groups at the peripheral positions, such an elemental composition could correspond to the* So*-NCC1 lacking the hydroxyl group at C8^2^, a structure not described so far (Table 1). To further characterize this compound, IT-MS^2^ was performed and the most prominent signals corresponding to similar product ions of* So*-NCC1 were observed, one at* m*/*z* 631 corresponding to the loss of H_2_O and one at* m*/*z* 492 corresponding to the loss of ring A, as can be observed in Figure 3(c) [21]. Consistency of the monoisotopic peaks and the related isotopic peaks was determined by high resolution ESI-qTOF-MS^2^ analyses, which obtained positive records for all product ions. With these data, the new NCC structure was tentatively assigned as the one presented in Table 2. This structure is described by first time in a vegetal tissue, and it presents some peculiar features in comparison with known chlorophyll metabolites. It is assumed that the first modification in the NCC structure (in fact the modification in the structures occurs at the precursor of NCC level, [30]) takes place at C8^2^, introducing an hydroxyl group that will later allow the esterification with other functional groups such as: glucopyranosil, malonyl or even O-malonyl-glucopyranoside units (Table 1). In fact, there are only two identified NCCs with an intact methyl group at C8^2^ (keeping the structural pattern of chlorophyll at that position):* Cj*-NCC2/*So*-NCC5 [1, 21], which is consequence of the direct modification from chlorophyll precursor, neither without any additional functional group nor at C3, C8^2^, or O13^4^. And the second one is* Bn*-NCC4/*At*-NCC5 [27] which presents the methyl ester function at O13^4^ as the chlorophyll precursor. Then,* Cl*-NCC3 is a very interesting compound because it shows peripheral substituents/modifications at C3 and O13^4^, but not at C8^2^. This means that all of the three positions are equally susceptible to experiment side reactions, and that the preferences for the NCC modifications could be a species-related matter.

The last chlorophyll breakdown product detected in ripened lemon fruits is denominated* Cl*-NCC4 (57.8 min, peak 4, Figure 1) with an exact mass determined by high resolution ESI-qTOF-MS of* m*/*z* 807.3415, corresponding to C_41_H_50_N_4_O_13_. This composition correlates with the NCC described in senescent tobacco leaves (*Nr*-NCC2, [22]), Arabidopsis leaves (*At*-NCC4, [25]), maize leaves (*Zm*-NCC2, [23]), and pears and apples fruits (*Pc*-NCC2 and* Md*-NCC2, [26]). Senescent leaves from maize were used as standard source, for chromatographic and mass spectrum comparison. The standard* Zm*-NCC2 from maize and the candidate from lemon fruit peels* Cl*-NCC4 coeluted at the same retention time. For further confirmation IT-MS^2^ analyses were performed, obtaining the same product ions both from the standard (*Zm*-NCC2) and from the* Cl*-NCC4 (Table 2):* m*/*z* 684, 645, 613, and 490 which correspond to the loss of ring A, the sugar moiety, the sugar moiety and MeOH, and ring A, the sugar moiety and MeOH, respectively. The IT-MS^2^ analysis is shown in Figure 3(d). All these product ions have been previously described for this NCC. Consistency of the observed product ions was checked by high-resolution ESI-qTOF-MS^2^ obtaining positive records for all product ions. Consequently,* Cl*-NCC4 is the equivalent of* Nr*-NCC2,* At*-NCC4,* Zm*-NCC2,* Pc*-NCC2, and* Md*-NCC2, in lemon fruit peels.

From all the enzymes responsible of the catabolism of chlorophylls to NCC/DNCC, only few of them have been identified. One is MES16 [28], which is able to deesterify the carboxymethyl group at O13^4^. Taking into account the NCC profile in lemon fruit peels (Table 2), the esterified compounds (*Cl*-NCC1,* Cl*-NCC2, and* Cl*-NCC4) are predominant; this implies that MES16 is not a very active enzyme in this fruit. However, the enzyme able to dihydroxylate the vinyl group at C3, an enzyme not yet identified, seems to be very important during the chlorophyll catabolism in lemon fruit, as the NCCs with dihydroxylated function at C3 are the main ones in the NCC profile of this fruit. Another aspect intriguing in lemon peels is that the only group that esterifies the OH group at C8^2^ is a glucopyranosil unit. It seems clear that the NCCs exhibit preferences in its peripheral substituents for a reason not completely explained yet. In the specific situation of the lemon fruits, the major availability of glucopyranosil groups or a higher specificity of the enzyme responsible for the esterification with glucopyranosil units could be a plausible reason. Anyway, further investigations are needed to understand the complete chlorophyll catabolism.

## 4. Conclusions

Here we exploited the high selectivity of high resolution ESI-qTOF MS data in combination with MS^2^-based reactions with the use of data postprocessing software algorithm capabilities considering accurate mass, isotopic pattern and MS^2^ fragmentation profile. This procedure accelerates analysis and determination of chlorophyll catabolites to show the heterogeneous profile of structures some of them not identified so far. Specifically, the identification of the NCC presents in the lemon fruit peels has allowed to conclude that the peripheral positions of NCC are equally susceptible to experiment side reactions, and that the preferences for the NCC modifications could be a species-related matter. In fact, the NCCs of the lemon fruits show preferences for the dihydroxylation at C3, the esterification with glucopyranosyl unit at C8^2^, and esterification at O13^4^.

## Figures and Tables

**Scheme 1:**
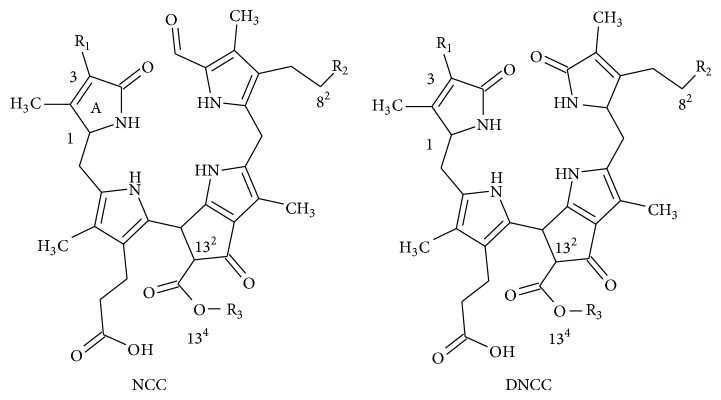


**Scheme 2:**
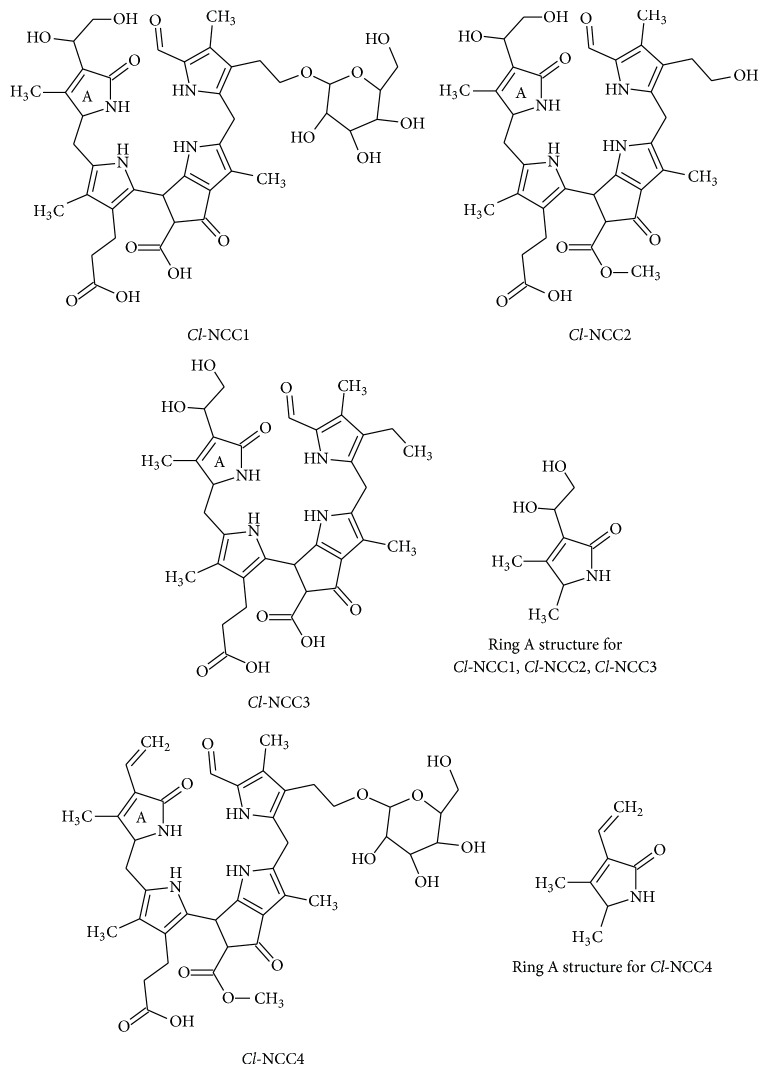


**Table 1:** *In-house* database of known NCCs and DNCCs built for screening chlorophyll catabolites in vegetal tissues.

	R_1_ at C3	R_2_ at C8^2^	R_3_ at O13^4^	M.W.	E.C.
**NCCs**					
*So*-NCC2/*Hv*-NCC1/*Ej*-NCC1/*Mc*-NCC42	CH(OH)-CH_2_OH	OH	CH_3_	678.2901	C_35_H_42_N_4_O_10_
*So*-NCC1	CH(OH)-CH_2_OH	OH	H	664.2744	C_34_H_40_N_4_O_10_
*Cj*-NCC1/*So*-NCC4/*Pc*-NCC1/*Md*-NCC1/ *Sw*-NCC58/*Lo*-NCC1/*Ls*-NCC1/*Ej*-NCC4/*Mc*-NCC61	CHCH_2_	OH	CH_3_	644.2846	C_35_H_40_N_4_O_8_
*So*-NCC3/*Bn*-NCC3/*At*-NCC2/*Ej*-NCC3/*Mc*-NCC49	CHCH_2_	OH	H	630.2690	C_34_H_38_N_4_O_8_
*Cj*-NCC2/*So*-NCC5	CHCH_2_	H	CH_3_	628.2897	C_35_H_40_N_4_O_7_
*Bn*-NCC4/*At*-NCC5	CHCH_2_	H	H	614.2741	C_34_H_38_N_4_O_7_
*Bn*-NCC1	CHCH_2_	O-Mal	H	716.2694	C_37_H_40_N_4_O_11_
*Nr*-NCC2/*Zm*-NCC2/*Pc*-NCC2/*Md*-NCC2/ *Tc*-NCC2/*At*-NCC4/*Mc*-NCC59	CHCH_2_	O-Glc	CH_3_	806.3374	C_41_H_50_N_4_O_13_
*Bn*-NCC2/*At*-NCC1	CHCH_2_	O-Glc	H	792.3218	C_40_H_48_N_4_O_13_
*Nr*-NCC1	CHCH_2_	O-(6′-O-Mal)-Glc	CH_3_	892.3378	C_44_H_52_N_4_O_16_
*Zm*-NCC1/*Tc*-NCC1/*Co*-NCC1	CH(OH)-CH_2_OH	O-Glc	CH_3_	840.3429	C_41_H_52_N_4_O_15_
*Co-*NCC2^a^	CH(OH)-CH_2_OH	O-Glc	H	827.3314	C_40_H_50_N_4_O_15_
*Ej*-NCC2^a^	CHCH_2_	O-Mal	CH_3_		
**DNCCs**					
*Hv*-DNCC1/*Ap*-DNCC1*/Co*-DNCC2	CH(OH)-CH_2_OH	OH	CH_3_	666.2901	C_34_H_42_N_4_O_10_
*At*-DNCC1	CHCH_2_	OH	H	780.3218	C_39_H_48_N_4_O_13_
*At*-DNCC6	CHCH_2_	H	CH_3_	616.2897	C_34_H_40_N_4_O_7_
*At*-DNCC3	CHCH_2_	OH	CH_3_	632.2846	C_34_H_40_N_4_O_8_
*At*-7HM-iso-DNCC5/*At*-9HM-DNCC4^b^	CHCH_2_	H	CH_3_	646.3003	C_35_H_42_N_4_O_8_
*Co*-DNCC1^a^	CH(OH)-CH_2_OH	O-Glc	CH_3_	828.3429	C_40_H_52_N_4_O_15_
*Ej*-DNCC4^a^	CHCH_2_	OH	CH_3_	794.3374	C_40_H_50_N_4_O_13_

M.W.: molecular weight. E.C.: elemental composition. The table is based on previously published results by Scherl et al. [24]. ^a^Compounds published by Ríos et al. [11, 12]. ^b^These compounds contain a hydroxymethyl function at C7 or C9.

See Scheme 1.

**Table 2:** Nonfluorescent chlorophyll catabolites composition in lemon fruit peels (*Citrus limon* L.) determined by HPLC/ESI-IT/high resolution qTOF-MS.

Compound	Peak number	*t*_*R*_ (min)	Time-of-flight					Ion trap
			Error (ppm)	mSigma	Molecular formula	MW. calc.	MW. meas.	Product ions (*m/z*)
*Cl*-NCC1	1	31.6	3.4	18.3	C_41_H_52_N_4_O_15_	841.3502	841.3473	809 [M-CH_3_OH+H]^+^ 684 [M-ring A+H]^+^ 679 [M-glucopyranosil+H]^+^

*Cl*-NCC2	2	39.5	3.1	8.3	C_35_H_42_N_4_O_10_	679.2974	679.2953	647 [M-CH_3_OH+H]^+^ 591 522 [M-ring A+H]^+^

*Cl*-NCC3	3	51.6	2.5	45.1	C_34_H_40_N_4_O_9_	649.2868	649.2852	631 [M-H_2_O+H]^+^ 492 [M-ring A+H]^+^

*Cl*-NCC4	4	57.8	4.0	16.7	C_41_H_50_N_4_O_13_	807.3447	807.3415	684 [M-ring A+H]^+^ 645 [M-C_6_H_10_O_5_+H]^+^ 613 [M-C_7_H_14_O_6_+H]^+^ 490 [M-ring A-C_7_H_14_O_6_+H]^+^

See Scheme 2.
